# Critically Examining the Person–Environment Relationship and Implications of Intersectionality for Participation in Children's Rehabilitation Services

**DOI:** 10.3389/fresc.2021.709977

**Published:** 2021-09-14

**Authors:** Meaghan Reitzel, Lori Letts, Briano Di Rezze, Michelle Phoenix

**Affiliations:** ^1^School of Rehabilitation Science, McMaster University, Hamilton, ON, Canada; ^2^CanChild Centre for Childhood Disability Research, McMaster University, Hamilton, ON, Canada; ^3^Bloorview Research Institute, Toronto, ON, Canada

**Keywords:** ICF, intersectionality theory, participation, personal factors, environmental factors, children's rehabilitation, childhood disability

## Abstract

Participation of children in rehabilitation services is associated with positive functional and developmental outcomes for children with disabilities. Participation in therapy is at risk when the personal and environmental contexts of a child create barriers to accessing services. The International Classification of Functioning, Disability and Health (ICF) provides a framework for conceptualizing the personal and environmental factors linked to a child. However, it does not facilitate critical examination of the person–environment relationship and its impact on participation in children's rehabilitation. This perspective study proposes the use of intersectionality theory as a critical framework in complement with the ICF to examine the impact of systemic inequities on the participation in therapy for children with disabilities. Clinicians are called to be critical allies working alongside children and families to advocate for inclusive participation in children's rehabilitation by identifying and transforming systemic inequities in service delivery.

## Introduction

The International Classification of Functioning, Disability and Health (ICF) holistically conceptualizes everyday functioning and disability across the interconnected domains of the following: body functions and structure, activity, and participation (1). The ICF defines participation as the “involvement in a life situation” (1, 2). Participation at home, school, and community has positive outcomes for child development and provides children the opportunity to develop skills required to support the transition into adulthood (3–6). Children with disabilities experience opportunity limitations and restrictions in participation when compared with their peers without disabilities (7–9). Given its association with improved developmental outcomes, understanding, measuring, and optimizing participation for children with disabilities is a common aim in children's rehabilitation (3, 6, 10–12). The ICF provides a common language to classify, understand, and study health and its related outcomes (1). Although the ICF can be used as a tool to raise awareness of necessary social change (1), it does not facilitate critical examination of how systemic health inequities are sustained by the uneven distribution of power and resources as well as dominant social practices (13, 14). Application of a critical lens could enhance the potential of the ICF to advance social change through the critical examination of the personal and environmental factors that can impact participation and health outcomes.

For children with disabilities, participation in rehabilitation is linked with positive functional outcomes (15). Participation in children's rehabilitation can be described as the active involvement of children and parents in all aspects of the therapeutic process (16). Goals related to optimizing life participation for children with disabilities can be a focus of rehabilitation services (17, 18). Given the improved developmental and participation outcomes associated with participation in rehabilitation services, it is critical that families who chose to engage in children's rehabilitation have adequate access to services available to them. Of notable concern, is when a family experiences barriers to accessing rehabilitation services as a result of factors such as the age of a child, parental stress, and culture or socio-economic status (SES) (19, 20). Participation in therapy may be limited when personal and environmental factors create barriers to service use. For example, language barriers and navigating unfamiliar health systems have been identified by immigrant mothers as barriers to accessing available services for their child with a disability (21), which would have negative implications for participation in children's rehabilitation. At a health systems level, families who experience barriers (e.g., transportation, working hours of parent) to consistently attend appointments may be systematically excluded from participating in rehabilitation by policies, which result in families being discharged after missing a specified number of visits (22). The personal and environmental factors should be considered with respect to the societal influences and systemic inequities that may limit therapy participation for some children and families (23). Systemic inequities are defined as disparities in health outcomes as a result of the uneven distribution of power, goods, and services (24, 25). This perspective study describes how an intersectional lens can be applied to critically examine the potential impact of systemic inequities on participation in therapy for children with disabilities.

Grounded in a biopsychosocial model of disability, the ICF acknowledges the impact of environmental and personal contextual factors on the experiences of the participation of individuals (1, 2, 26, 27). In the ICF, the term environment is used broadly to represent the physical, social, attitudinal, and institutional context in which a person is situated (1, 2, 12, 28). The environment has been demonstrated to influence experiences of participation, with the potential to act both as a facilitator or barrier to participation for children with disabilities (6, 7, 28, 29). Parents of children with disabilities described features of the environment as making it harder for their children to participate in community-based activities (7), including participation in rehabilitation services. The potential influence of the social environment on participation was demonstrated in a systematic review examining the impact of family factors such as family structure, socio-demographic factors, parental behavior, and family resources on participation outcomes for children with disabilities, both generally and with specific reference to accessing the rehabilitation services of children (19, 29).

The ICF framework identifies personal factors that have been shown to affect participation such as gender, age, and ethnicity (1, 2). These personal factors contribute to the make-up of the unique identity of a person and are distinct from the disability or health condition (1). Individual categories of personal factors are not defined in the ICF (1). Examining the need for more specificity within the ICF personal factors has been identified in the literature (30, 31); however there is concern that classifying personal factors with single categorical distinctions risks discrimination and misrepresenting the personal factors with which a person does or does not identify (32–34). When single-identity categories (e.g., age, gender, or ethnicity) are considered in isolation service providers may lack sufficient information to set tailored goals collaboratively with clients, risk-making assumptions about how clients position themselves in relation to their personal factors and underestimate the environmental opportunities and challenges that may impact participation resulting from the person-environment relationship. Therefore, this paper proposes intersectionality theory as a means of bridging the understanding of the ICF personal and environmental factors that can impact participation in therapy. By applying an intersectional lens, all facets of the identity of a child are considered simultaneously, with specific acknowledgment for the environmental context in which a child with a disability is situated.

Intersectionality theory can be used to holistically identify and critically examine the aspects of identity by exploring the relationships that exist between facets of the identity of an individual (i.e., ICF personal factors) and the larger societal systems in which a person participates (35). Intersectionality allows for the application of a critical lens to examine how the ICF personal factors as experienced by a child with a disability contribute to whether society views them as belonging to groups of socially-perceived advantage or disadvantage. This social construction of the identity and place of a child in the society has implications for their participation in therapy. It is important to note that in this context the term critical is used to describe the process of thinking deeply about the intended and unintended consequences associated with our actions (36). Intersectionality theory explains that identity cannot be understood by examining individual elements of identity (37). Instead, we examine the socially constructed privilege and oppression associated with the interaction between multiple aspects of the identity of an individual and their environments (37, 38). Literature and frameworks related to childhood disability and participation illustrate the relationship between factors external to the child (ICF environment factors), factors internal to the child (ICF personal factors), and participation outcomes (12, 26, 28). The family of participation-related constructs model embeds participation within the surrounding environmental contexts and makes explicit the bidirectional relationship between factors intrinsic to an individual and participation (28). In childhood disability literature, next steps should include a critical exploration of how the personal factors of an individual are privileged or oppressed, impacting their life participation. For example, it is understood in the literature that the environmental context mediates participation frequency and level of involvement in activities (6). Given that many personal factors cannot be changed, modifying the environment has been discussed as an approach to facilitate participation (6). Critically examining the social, attitudinal, and institutional environments in which children with disabilities are situated creates opportunities to identify barriers to life participation. As a result, participation-enhancing solutions, focused on modifying the systemic environmental context in which inequities exist, can be developed. Intersectionality theory provides the critical lens needed to examine contextual factors identified using the ICF to examine potential systemic inequities impacting participation.

## Critically Examining Participation in Childhood Disability

Children with disabilities have unique identities, in part, shaped by the physical, social, attitudinal, and institutional environments around them (39). The social environment referenced in the ICF includes the family of a child. Parents are the most proximal environment to a child, playing a critical role in facilitating opportunities for participation and providing care for children with disabilities. However, the broader environments in which the family and child are situated need to be considered (40) to understand the implications for participation in children's rehabilitation. Personal factors such as age, sex, or ethnicity do not alone determine therapy participation. Instead, implications for the participation of a child arise when the interplay between their unique personal factors and the broader environmental context results in experiences of systemic inequities such as ageism, sexism, racism, or ableism. Experiences of discrimination risk limiting a child with a disability from fully participating in important aspects of their lives, including rehabilitation therapies. Applying a critical intersectional lens to participation in therapy allows clinicians to holistically consider how the identity of a child interacts with the surrounding environment to better understand implications for participation. Opportunities for participation in therapy are created or repressed according to the complex interaction between a the personal identity factors and the systemic inequities of a child that exist in rehabilitation environments.

Examining the personal factors of a child individually does not provide an adequate foundation for understanding implications for participation in pediatric rehabilitation services. Applying an intersectional lens allows us to critically consider how personal factors, as outlined by the ICF, interact with the ICF environmental domains to maintain systemic inequities and impact participation in therapy. This creates an opportunity for the rich examination of potential facilitators and barriers to participation. As an example, let us briefly explore the personal factors of sex and culture in relation to disability. In some developing countries there continues to be a gap in participation opportunities between boys and girls in life activities such as schooling and employment (41–43). Additionally in some cultures, stigmatization of disability prevents parents from seeking therapy services for a child with a disability (44, 45). In this context, opportunities for participation, including participation in therapy, for a girl with a disability may be limited due to the possibility of sexist and ableist discrimination resulting from person–environment interactions. This example demonstrates the need to explore the intersection between the personal factors of sex and culture as influenced by disability and the environment in which the family is situated to understand implications for participation in therapy. This example illustrates how intersectionality theory can be applied in complement to the ICF by contributing a critical lens to examine the interaction between the ICF personal (i.e., sex, culture) and the environmental factors (i.e., stigma) to identify potential participation restrictions resulting from systemic discrimination. Although this study focuses on implications for participation in therapy, this perspective can be applied when examining how children with disabilities participate in a variety of life contexts.

## Discussion

Applying a critical perspective facilitates an in depth understanding of how the person–environment relationship potentially impacts the participation of children with disabilities in rehabilitation services. But what do we as childhood disability clinicians do with the insights gleaned from critically examining the interplay between the intersectional identity and surrounding environment of a child? How can this information be used to optimize participation and inclusion of this population in childhood rehabilitation? By considering how the personal and environmental context of a child might impact their participation in therapy, clinicians have the opportunity to conceptualize solutions to enhance access for an individual family as well as identify patterns in participation limitations that could drive system-level change. At the clinical practice level, the use of a family-centered and solution-focused approach, whereby the family is actively engaged as collaborators in therapy, may be a way for clinicians to gain an understanding of how the person–environment relationship creates barriers to therapy participation and involve families in developing solutions (46, 47). A family-centered approach to care recognizes the expert knowledge of a parent about their child and has been associated with improved access and health outcomes in children with special needs (47, 48). Therefore, a family-centered approach to identifying and co-creating individualized solutions addressing parent-identified barriers to participation in children's rehabilitation is recommended. Inclusion of diverse stakeholders has been identified as critical in policy development (49). Clinicians can advocate for the representation of the family voice in the development of policy that supports inclusive participation in therapy.

As a critical approach, intersectionality seeks not only to understand lived experience of others and highlight oppression, it also aims to generate new knowledge that calls for change to inequitable social practices (37, 50–52). Clinicians are well positioned to become allies, working alongside families and children to understand their experiences of being included and excluded from opportunities to participate in therapy and identify potential inequities resulting from the person–environment relationship. This information provides a platform to highlight the role of society in facilitating or hindering participation in rehabilitation services and advocate for system-level changes, such as resource allocation, program, and service design or policy reform that optimize inclusion. The discomfort clinicians may experience while acting as critical allies is important to acknowledge. On one hand, equity is a core component of health ethics and should be advocated for (14, 53) but on the other hand, clinicians have a commitment to follow the rules and practices of the health system by which they are employed. Critical allyship may require clinicians to advocate in opposition to the dominant policies and practices of the system they work in. Although there is no clear solution to address the paradox created by critical allyship, clinicians can make use of frameworks such as the 7-step framework for critical analysis to reflect on the impact of their actions in practice and consider the potential harms and benefits associated with different courses of action (36).

There is a need for careful consideration on how clinicians align themselves and act upon this advocacy role to avoid employing a disempowering approach aiming to “fix” those in a position of socially constructed disadvantage (i.e., children with disabilities) (54). In the coin model of privilege and critical allyship, Nixon (2019) describes “practicing critical allyship” as an approach for individuals in a position of privilege (i.e., clinicians) to work in partnership with those experiencing oppression to identify and take action on the systems perpetuating inequities (i.e., restrictions in therapy participation). Critical allyship calls individuals in positions of power to acknowledge their experiences of privilege and how their advantaged position may contribute to sustaining dominant inequitable social practices (54). As critical allies, clinicians can learn from parents and children about their experiences to better understand the impact of system inequities on participation in children's rehabilitation (54). Additionally, under the guidance of parents and children, clinicians can use their privileged positioning to advocate for system change among other power-privileged groups (i.e., health service decision makers) (54).

By applying an intersectional lens to examine the impact of the person–environment relationship on participation in therapy for children with disabilities, clinicians have the opportunity to practice critical allyship alongside children and parents to transform inequitable systems. However, applying a critical lens to participation in therapy may be a new approach for some clinicians. How as a group of professionals do we implement and become comfortable critically examining participation outcomes for children with disabilities? We suggest looking toward the ICF as a framework to get started. The ICF is a well-recognized, familiar, and frequently referenced framework in the childhood disability literature (26, 55–57). Through listening to the thoughts and feelings shared by families of children with disabilities, clinicians can apply the ICF to conceptualize the personal and environmental factors relevant to the individualized family context. Literature is available to provide clinicians with pragmatic guidance for integrating the ICF into their practice (58). Critical examination of how the dominant ways of thinking in our society impact participation in therapy is currently under-represented in the literature. An intersectional lens can be used in complement the ICF to critically examine contextual factors, identify barriers and facilitators to participation in children's rehabilitation, and create actionable change toward more inclusive systems.

## Data Availability Statement

The original contributions presented in the study are included in the article, further inquiries can be directed to the corresponding author/s.

## Author Contributions

MR, LL, BD, and MP contributed to the conception of this work, contributed comments and edits to the initial and subsequent versions of the manuscript, read, and approved the final manuscript. MR wrote the first draft of the manuscript. MP is the senior author of this manuscript. All authors contributed to the article and approved the submitted version.

## Funding

Funding for publishing this open access article was obtained through of the McMaster University School of Rehabilitation Science Dr. Ian and Shirley Rowe Scholarship 2021–2022 awarded to the first author (MR).

## Conflict of Interest

The authors declare that the research was conducted in the absence of any commercial or financial relationships that could be construed as a potential conflict of interest.

## Publisher's Note

All claims expressed in this article are solely those of the authors and do not necessarily represent those of their affiliated organizations, or those of the publisher, the editors and the reviewers. Any product that may be evaluated in this article, or claim that may be made by its manufacturer, is not guaranteed or endorsed by the publisher.
